# Effects of different dosages/frequency of Xuebijing injection (a Chinese patent) for sepsis: a network meta-analysis of randomized controlled trials

**DOI:** 10.3389/fmed.2025.1577414

**Published:** 2025-05-22

**Authors:** Guangyan Liu, Xueping Qu, Ruowen Jiang, Yongxing Wu, Zan Qin, Hui Zhang, Ruze Ma, Jiawei Xue, Junwu Wang, Xueqin Xu, Chenxi Yan, Xiaodan Wei, Litao Guo

**Affiliations:** ^1^Department of Critical Care Medicine, The First Affiliated Hospital of Xi'an Jiaotong University, Xi'an, China; ^2^Shaanxi Key Laboratory for Sepsis Research, Xi'an, China

**Keywords:** sepsis, Chinese herbal medicines, Xuebijing injection, network meta-analysis, randomized controlled trial

## Abstract

**Objective:**

Xuebijing injection (XBJ) has been widely recognized in the treatment of sepsis, however, inadequate information regarding XBJ's optimal dosage and frequency suffice. We aimed to assess the effectiveness of various doses and administration frequencies in patients with sepsis using a network meta-analysis (NMA) to offer therapeutic prescription guidance.

**Methods:**

We examined eight databases for 1,765 randomized controlled trials published before July 2024, organized the literature using NoteExpress software and extracted data using Microsoft Excel software. The literature's quality was assessed using the risk of bias evaluation approach endorsed by the Cochrane Collaboration. The analysis was conducted by NMA inside a frequency-based framework.

**Results:**

Forty-three qualifying studies were included in the analysis, including 5,818 participants. Regarding the enhancement of 28-day mortality, 50 Milliliter (ml)-tie in die (tid) exhibited optimal efficacy, 100 ml-tid demonstrated superior efficacy in ameliorating APACHE II scores, 50 ml-bis in die (bid) proved more effective in enhancing the activated partial thromboplastin time (APTT), while 100 ml-quaque die (qd) significantly improved C-reactive protein (CRP) levels. Additional findings are displayed in net league tables, forest plots, and funnel plot.

**Conclusions:**

A daily dose of 100 ml of XBJ was associated with improvement in APTT and CRP levels in patients with sepsis, a daily dose of 150 ml may decrease 28-day mortality; while XBJ with a single-day dose of 300 ml is more effective at improving the APACHE II score, higher dosages correlated with improved prognosis in these patients compared to other doses.

## 1 Introduction

Sepsis is a critical condition characterized by organ dysfunction resulting from the body's aberrant response to infection (1). As global awareness of sepsis has increased, it is estimated that 6–9 million individuals worldwide develop it annually (2). In the United States, Sepsis is the primary cause of mortality among patients with critical sickness, resulting in about 210,000 fatalities per year (3). Patients recovering from sepsis are frequently readmitted due to organ dysfunction (4) and the emergence of new symptoms (5). Sepsis-related rehospitalization constitutes 12.2% of all hospital readmissions in the United States (6). A study from China reports that the morbidity rate of sepsis in the Intensive Care Unit (ICU) was 20.6%, the mortality rate was 35.5%, and the mortality rate for severe sepsis exceeded 50% (7). The management of sepsis presents certain challenges. The potential for substantial advancements in early prevention, pharmacological treatment, lifesaving measures, and rehabilitation to reduce the current high rates of morbidity, mortality, and rehospitalization is a critical issue that should be addressed to enhance critical care, emergency medicine, and preventive medicine in future (8).

Research has shown that certain Chinese herbal medicines can influence inflammation and the immune response. Additionally, the combination of herbal medicines with antibiotics has been found to decrease the prevalence of drug-resistant bacteria and the incidence of multiple organ dysfunction syndrome (MODS). In the Chinese treatment guidelines and expert consensus (9–11). for sepsis management, herbal medicines are recommended as complementary to conventional sepsis treatment, with XueBiJing injection (XBJ) being a widely used injectable product authorized in China since 2004 for the treatment of Sepsis and MODS. XBJ was formulated from a blend of Carthamus tinctorius flowers (Honghua), Paeonia lactiflora roots (Chishao), Salvia miltiorrhiza roots (Danshen), Ligusticum chuanxiong rhizomes (Chuanxiong), and Angelica sinensis roots (Danggui) (12). Several meta-analyses (MA) have shown that combining XBJ with the standard treatment for sepsis can further reduce mortality by 28 days, reduce complication rates, and enhance patient prognosis (13–15).

**Figure 1:**
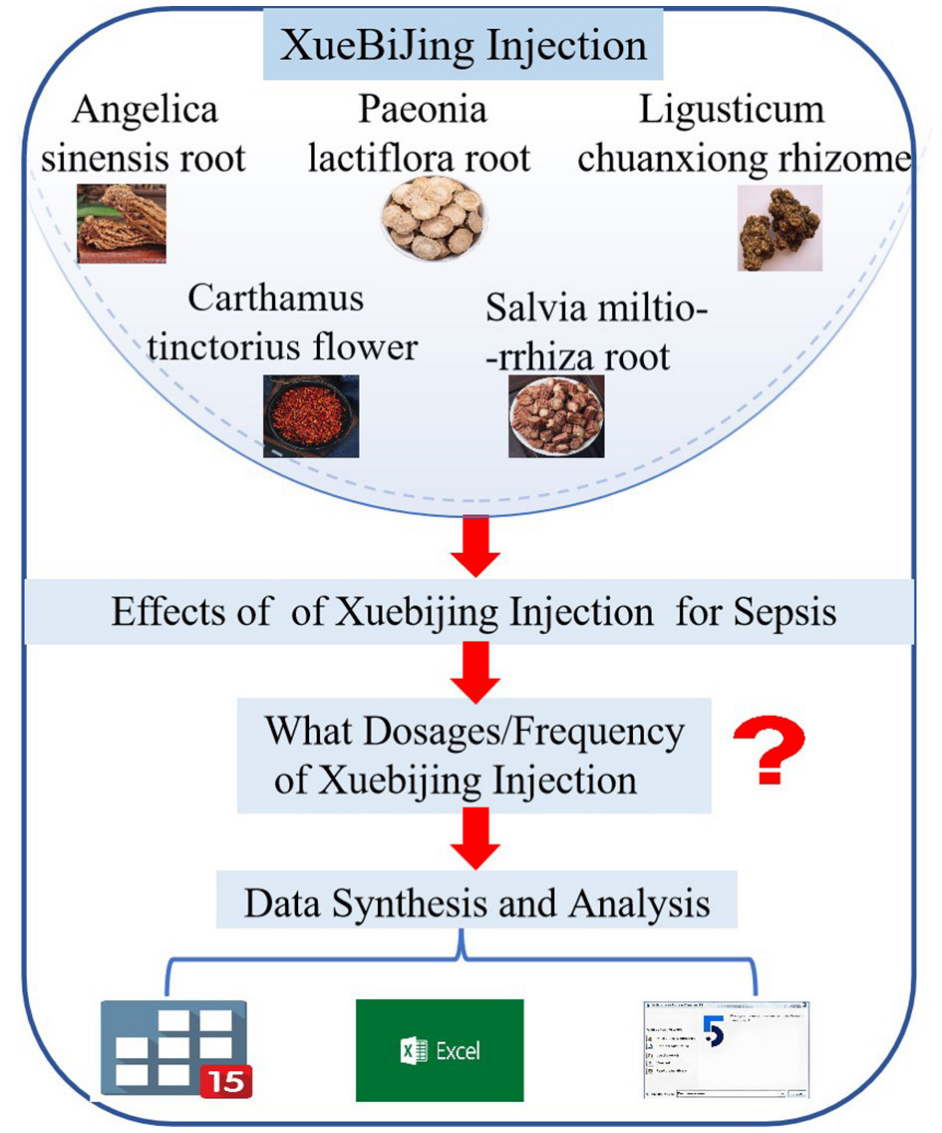
Cognitive process diagram.

Nonetheless, in clinical applications and associated studies, the dosage and frequency of XBJ differs (16, 17), and no study has clarified the optimal dosage and frequency of XBJ for sepsis treatment to enhance patient prognosis and reduce mortality. This study presents preliminary findings on the appropriate dosage and frequency of XBJ using network meta-analysis (NMA), aiming to guide doctors and data support for future research on XBJ as an adjunctive therapy for sepsis, as shown in Figure 1.

## 2 Methods

This study adheres to the PRISMA-2020 statement (18). The trial was not registered, and a protocol was not established.

### 2.1 Data sources and search strategy

We conducted a comprehensive search across many academic databases, including the China National Knowledge Infrastructure (CNKI), WanFang Medical Database, China Science and Technology Journal Database (VIP), Chinese Biomedical Literature Database (CBM), PubMed, Embase, Web of Science, and Cochrane Library. The literature search included studies that compared XBJ with placebo or Standard drug therapy (STDT) in randomized controlled trials (RCTs), with publications collected up to July 2024. In addition, we conducted a thorough manual examination of the pertinent MAs, reviews, pooled analyses, and reference lists of the included studies to ensure that no significant information was inadvertently excluded. A comprehensive literature search encompassing the topic “Sepsis” was equally performed. Specifically, we focused on the RCTs that investigated the efficacy and safety of XBJ.

### 2.2 Study selection and selection criteria

Two investigators independently performed the literature search, and in cases of disagreement, a group discussion was held to reach a consensus. Inclusion criteria were as follows: (1) compliance with an RCT, (2) the participants should be diagnosed with sepsis, based on “The Third International Consensus Definitions for Sepsis and Septic Shock (Sepsis-3) (1),” and (3) no restrictions were placed on gender, age, geographic location, ethnicity, race, duration of disease. Exclusion criteria included: (1) non-RCTs, such as animal experimentations, reviews, systematic evaluations, case reports, or conference abstracts, (2) non-septic patients or subjects with conditions other than sepsis, and (3) duplicate publications, plagiarism, studies with unextractable or controversial data.

### 2.3 Data extraction and quality assessment

Four authors (XP Q, RW J, Z H, and Q Z) screened and extracted data using NoteExpress software for literature screening and management. The software excluded duplicates, animal experiments, MAs, case reports, and other irrelevant materials. Subsequently, two authors reviewed the titles and abstracts to identify compliant studies and non-compliant studies were excluded. Thereafter, the literature was acquired and comprehensively examined to encompass studies that satisfied the specified criteria. In instances of disagreement, a neutral third party was involved to facilitate resolution. The extracted data included the first author, publication year, country, ethnicity, sample size, duration of treatment, application of anti-heart failure drugs or glucose-lowering pharmaceuticals, outcomes, and methods of randomization and blinding.

The quality of the studies was assessed using the Cochrane Collaboration's risk-of-bias evaluation approach. To construct the Literature Quality Assessment Form, the RevMan 5.4 software was utilized. Any disagreements between RW J and XP Q were reevaluated and discussed until a consensus was reached.

### 2.4 Data synthesis and analysis

NMA is a collective term for indirect and mixed treatment comparisons, and one key advantage is its ability to quantitatively compare various interventions for a given disease. Additionally, it allows the integration of evidence from both direct and indirect comparisons. This enabled the ranking of interventions based on their superiority or inferiority in terms of specific outcomes, thereby facilitating the identification of the optimal option. The majority of NMA was conducted using frequency-based or Bayesian methods. Following the process of extracting data and evaluating the quality of the studies that were included, the collected data were subjected to frequency-based NMA using Stata15 software. Network plots were generated using the same software to summarize the evidence from the direct and indirect comparisons. Additionally, the surface under the cumulative ranking (SUCRA) was computed to rank the interventions for each outcome, assigning scores ranging from 0 to 100%. The forest plot displays the findings of the studies included in the analysis, comparing them with a placebo group across five different outcome measures. Publication bias was evaluated by visually examining the funnel plots of the five outcomes. The net league table (inverted triangle plot) graphically presents the results of mixed evidence reporting for pairwise comparisons of different interventions, with a statistically significant difference (*P* < 0.05) between the experimental and control groups when the 95% confidence interval (CI) did not include a value of 1. The same software was used to conduct sensitivity analyses.

## 3 Results

### 3.1 Literature screening results

**Figure 2:**
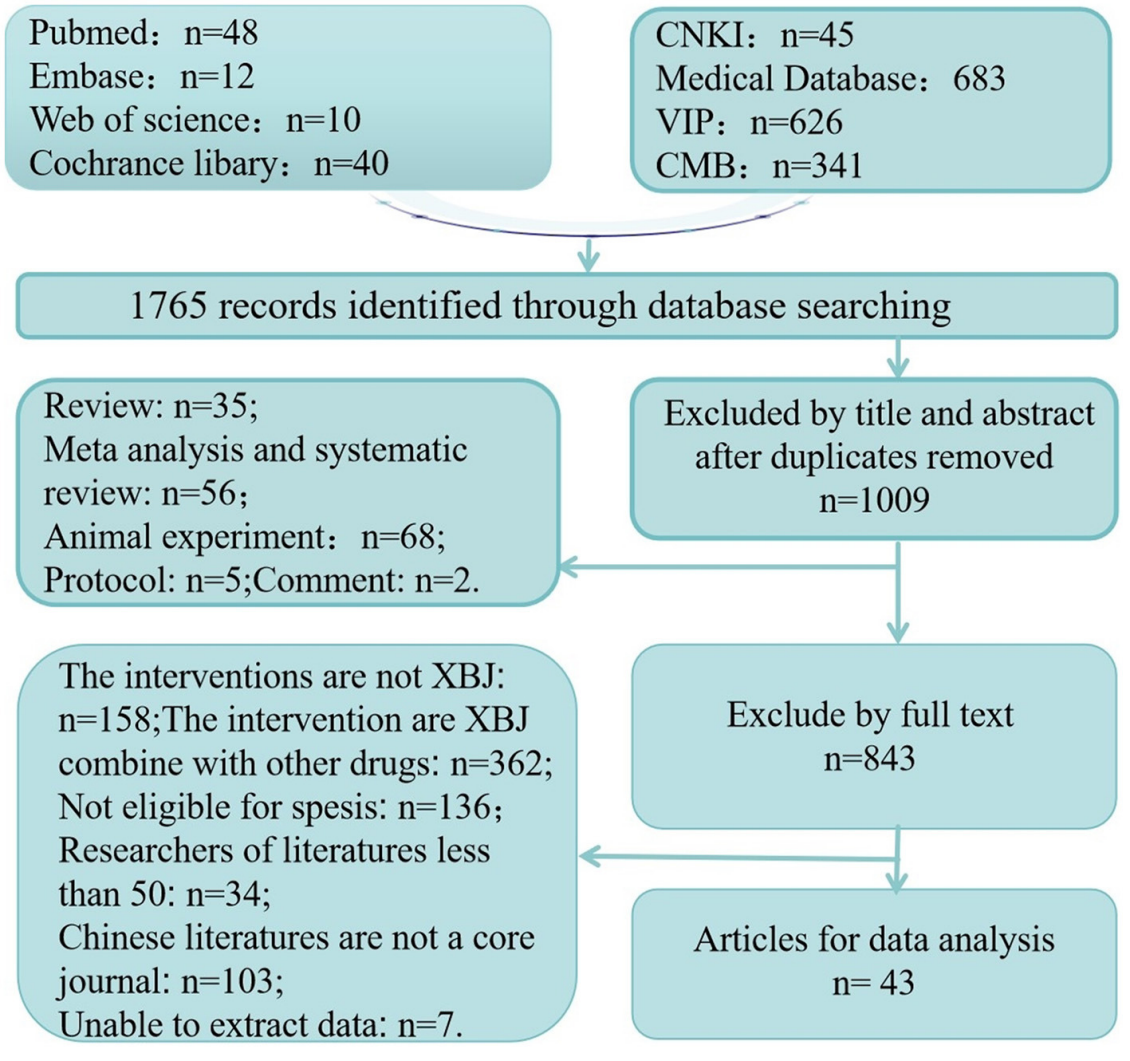
Study selection process.

Overall, 1,765 studies were identified. After excluding 756, duplicates 1,009 studies remained. Of these, 43 studies met the inclusion criteria. Detailed information regarding the literature screening process is shown in Figure 2.

### 3.2 Study characteristics

The baseline information of all included studies is presented in Supplementary Appendix S1. The treatment duration ranged from a minimum of 3 days (19, 20) to a maximum of 2 weeks (21–27). Seven studies used a 12 h dosing interval, classified as “bid” (16, 19, 20, 28–31), while three studies used an 8 h interval and were included as “tid” (19, 22, 32). The largest sample size among the studies was 1,817 patients (16), and the smallest sample size was 62 patients (33). This study included 59% male and 38% female participants, although some studies did not report the exact breakdown of male and female participants (34). The treatment group received 10 interventions that varied in dosage and frequency. Control group interventions included placebo and standard drug therapy (STDT). The primary endpoints of the studies were assessed using four outcome metrics, with detailed data for each outcome metric shown in Table 1.

**Table 1:** Baseline information for data analysis of included studies.

**Study**	**Treatments**	**28-d mortality (r/n)**	**APACHE II score (Mean ±SD)**	**APTT (Mean ±SD)**	**CRP (Mean ±SD)**
Cheng et al. (19)	50 ml-bid	NA	12.41 ± 5.13	NA	NA
	PLA		17.03 ± 6.82		
Chen et al. (60)	100 ml-tid (3d)	NA	NA	NA	115.07 ± 13.86
	100 ml-bid (3d)				120.37 ± 30.43
	STDT (3d)				129.30 ± 19.96
	100 ml-tid (7d)				93.43 ± 10.41
	100 ml-bid (7d)				102.03 ± 13.98
	STDT (7d)				109.47 ± 7.08
Dai et al. (35)	100 ml-bid	NA	12.57 ± 1.57	23.46 ± 2.31	85.47 ± 12.34
	STDT		17.23 ± 2.03	28.12 ± 2.65	100.38 ± 15.32
Dong et al. (33)	100 ml-bid	NA	11.23 ± 2.17	NA	NA
	STDT		14.45 ± 3.24		
Dou et al. (36)	100 ml-bid	10/45	NA	NA	NA
	STDT	12/46			
Gong et al. (37)	50 ml-bid	1/42	6.62 ± 2.91	30.95 ± 8.48	NA
	STDT	1/40	12.87 ± 4.54	42.25 ± 7.73	
Ji et al. (38)	100 ml-tid	NA	6.03 ± 1.55	34.16 ± 6.38	10.73 ± 3.85
	STDT		9.46 ± 2.58	37.33 ± 6.86	14.98 ± 5.64
Ji et al. (39)	50 ml-bid	NA	13.25 ± 1.36	NA	NA
	STDT		15.13 ± 2.11		
Jia and Bao (20)	100 ml-bid (7d)	NA	13.63 ± 2.64	NA	35.64 ± 11.67
	STDT (7d)		15.46 ± 3.75		90.65 ± 16.57
	100 ml-bid (3d)		NA		68.59 ± 17.23
	STDT (3d)				102.43 ± 18.42
Jiang et al. (40)	50 ml-bid	12/95	9.48 ± 2.85	NA	NA
	PLA	15/95	11.9 ± 3.32		
Jiang et al. (28)	60 ml-bid	NA	10.7 ± 2.6	NA	NA
	STDT		13.8 ± 1.3		
Jiang (48)	50 ml-bid	NA	NA	30.36 ± 5.34	NA
	PLA			45.83 ± 6.71	
Li et al. (21)	100 ml-bid	NA	NA	35.56 ± 5.56	NA
	STDT			25.53 ± 3.83	
Liu (41)	100 ml-qd	9/34	13.81 ± 7.53	NA	78.85 ± 48.78
	STDT	13/30	15.36 ± 8.74		96.59 ± 81.01
Liu et al. (16)	100 ml-bid	165/878	NA	NA	NA
	PLA	230/882			
Liu et al. (29)	50 ml-bid	NA	13 ± 2.5	30.5 ± 6.4	NA
	STDT		15.2 ± 3.7	34.7 ± 6.2	
Lu et al. (50)	50 ml-bid	NA	8.42 ± 3.11	NA	NA
	STDT		11.1 ± 2.85		
Ma et al. (51)	50 ml-bid	NA	NA	32.18 ± 6.31	NA
	STDT			36.49 ± 6.78	
Ming et al. (52)	300 ml-qd	NA	NA	NA	16.15 ± 3.12
	STDT				28.65 ± 4.52
Pu et al. (22)	50 ml-tid (7 d)	NA	13.01 ± 2.61	NA	NA
	STDT (7 d)		14.15 ± 2.66		
	50 ml-tid (14 d)	5/45	11.22 ± 2.15		
	STDT (14 d)	13/45	12.28 ± 2.73		
Shao et al. (53)	50 ml-bid	NA	9.4 ±5.4	NA	NA
	STDT		14.8 ± 5.7		
Shao et al. (32)	100 ml-bid	NA	NA	26.26 ± 5.86	NA
	STDT			38.05 ± 8.56	
Shen et al. (23)	100 ml-bid	NA	NA	NA	32.39 ± 6.25
	STDT				41.28 ± 7.39
Shi et al. (42)	100 ml-qd	NA	8.29 ± 5.62	NA	NA
	STDT		11.39 ± 6.13		
Song et al. (54)	50 ml-bid	1/47	10.4 ± 1.1	NA	NA
	STDT	3/47	15.3 ± 1.4		
Su et al. (43)	100 ml-bid	NA	NA	NA	53.7 ± 18.8
	STDT				91.3 ± 32.8
Sun and Yang (30)	100 ml-bid	NA	NA	35.5 ± 1.11	NA
	STDT			39.82 ± 0.48	
Sun (44)	50 ml-bid	NA	6.29 ± 1.71	NA	NA
	STDT		8.35 ± 1.82		
Wang et al. (24)	100 ml-qd (7 d)	NA	NA	NA	71.22 ± 40.75
	STDT (7 d)				105.68 ± 47.31
	100 ml-qd (14d)				37.0 ± 33.47
	STDT (14d)				76.47 ± 59.04
Wang et al. (55)	100 ml-qd	NA	13.61 ± 7.62	NA	NA
	STDT		16.34 ± 8.7		
Wang et al. (45)	100 ml-bid	NA	8.58 ± 1.64	NA	NA
	PLA		10.02 ± 1.98		
Wu et al. (25)	50 ml-bid	NA	NA	NA	77.1 ± 24.9
	STDT				97.5 ± 27.6
Qiao (31)	50 ml-bid	NA	11.3 ± 2.8	NA	NA
	STDT		13.6 ± 3.1		
Xing et al. (56)	100 ml-bid (7 d)	NA	8.93 ± 5.5	NA	NA
	STDT (7 d)		12.99 ± 6.03		
	100 ml-bid (10 d)	9/33	8.12 ± 4.36		
	STDT (10 d)	16/30	12.49 ± 5.97		
Xu et al. (26)	50 ml-bid	NA	NA	NA	28.5 ± 20.0
	STDT				45.4 ± 19.6
Yang (49)	50 ml-bid	NA	12.92 ± 3.13	31.98 ± 6.98	NA
	STDT		16.17 ± 4.89	42.19 ± 7.12	
Yin and Li (57)	100 ml-bid	18/88	NA	NA	NA
	STDT	29/83			
Zhang and Pei (46)	100 ml-bid	NA	NA	29.41 ± 4.72	NA
	STDT			32.64 ± 3.61	
Zhang et al. (34)	50 ml-bid	NA	NA	39.47 ± 4.73	NA
	STDT			42.75 ± 5.24	
Zhang (27)	100 ml-bid	NA	8.72 ± 6.23	NA	27.38 ± 22.13
	STDT		11.59 ± 5.66		35.76 ± 24.66
Zhang et al. (59)	50 ml-bid	NA	12.5 ± 3.16	37.88 ± 3.95	NA
	STDT		15.63 ± 2.79	40.12 ± 4.08	
Zhang et al. (47)	50 ml-bid (7d)	NA	14.02 ± 5.71	NA	NA
	STDT (7d)		17.42 ± 6.23		
	50 ml-bid (10d)		11.06 ± 4.02		
	STDT (10d)		13.19 ± 4.17		
Zhu et al. (58)	100 ml-bid	12/33	NA	NA	NA
	PLA	17/33			

### 3.3 Study quality assessment

**Figure 3:**
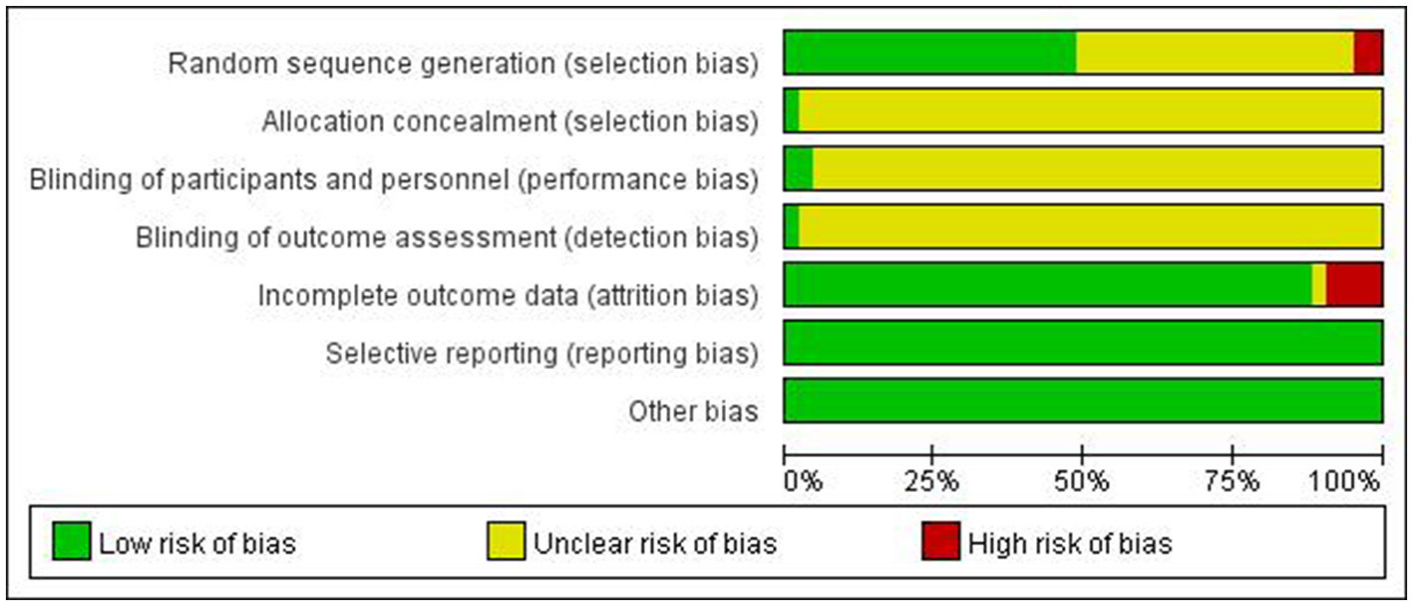
Risk of bias graph.

The quality of included studies was assessed using three categories: high, low, and unclear risk-of-bias. Considered factors included: (1) Selection bias- generation of randomized sequences: 19 studies used the random number table method (19, 20, 29, 33–47) one study used drawing lots (48), which was rated as “low bias,” one research used the order of admission time (49), which was rated as “high bias”, 20 studies mentioned randomization (16, 22–28, 30–32, 34, 47, 50–56), incapable of detecting if it was classified as “low bias” or “high bias,” and was rated as “unclear,” (2) Allocation concealment: none of the documents mentioned whether or not the allocation scheme was concealed, and it was rated as “unclear,” (3) Performance bias—investigator and subject blinding: two studies (16, 21) mentioned blinding and were rated as “low bias,” the remaining studies did not describe blinding and were rated as “unclear,” (4) Measurement bias—blinding of study outcomes: none of the papers mentioned whether blinding was conducted on the outcomes, and they were rated as “unclear,” (5) Attrition bias—totality of outcome data: the research outcomes of the four articles contain missing data (16, 30, 33, 46) were rated as “high bias,” other literatures did not include discharges, withdrawals due to adverse effects, etc., the rating was “low bias,” (6) Reporting bias—selective or insufficient reporting of outcome indicators: all outcome indicators of all studies were reported in total, and they were rated as “low bias,” (7) Other bias—the existence of other bias: none of the studies mentioned the existence of other bias, and it was rated as “low bias.” Refer to Figure 3.

### 3.4 Data synthesis and analysis

Global and local inconsistency tests were conducted on the four outcome studies using Stata15 software. A *P* > 0.05 indicated inconsistency between the direct and indirect comparisons, and the data were analyzed using the consistency model. The *P*-value for the local inconsistency test was computed using node-splitting analysis. If the resulting *P*-value exceeded 0.05, this indicated statistical consistency between the direct and indirect evidence. The data displayed in Table 2 suggest an inconsistency between the direct and indirect comparisons for the cardiovascular mortality outcomes. The *P*-values obtained from both the global and local inconsistency tests for all remaining outcome indicators were greater than the significance level of 0.05, indicating that there was no inconsistency between direct and indirect comparisons.

**Table 2:** The global inconsistency test and local inconsistency test for four outcomes.

**Outcomes**	**Global inconsistency (*P*-value)**	**Local inconsistency (*P*-value)**
28-d mortality	0.6201	> 0.1
APACHE II score	0.2154	> 0.1
APTT	0.6403	> 0.1
CRP	0.1313	> 0.1

APTT, activated partial thromboplastin time; CRP, C-reactive protein.

### 3.5 Network meta-analysis results

#### 3.5.1 The 28-day mortality

**Figure 4:**
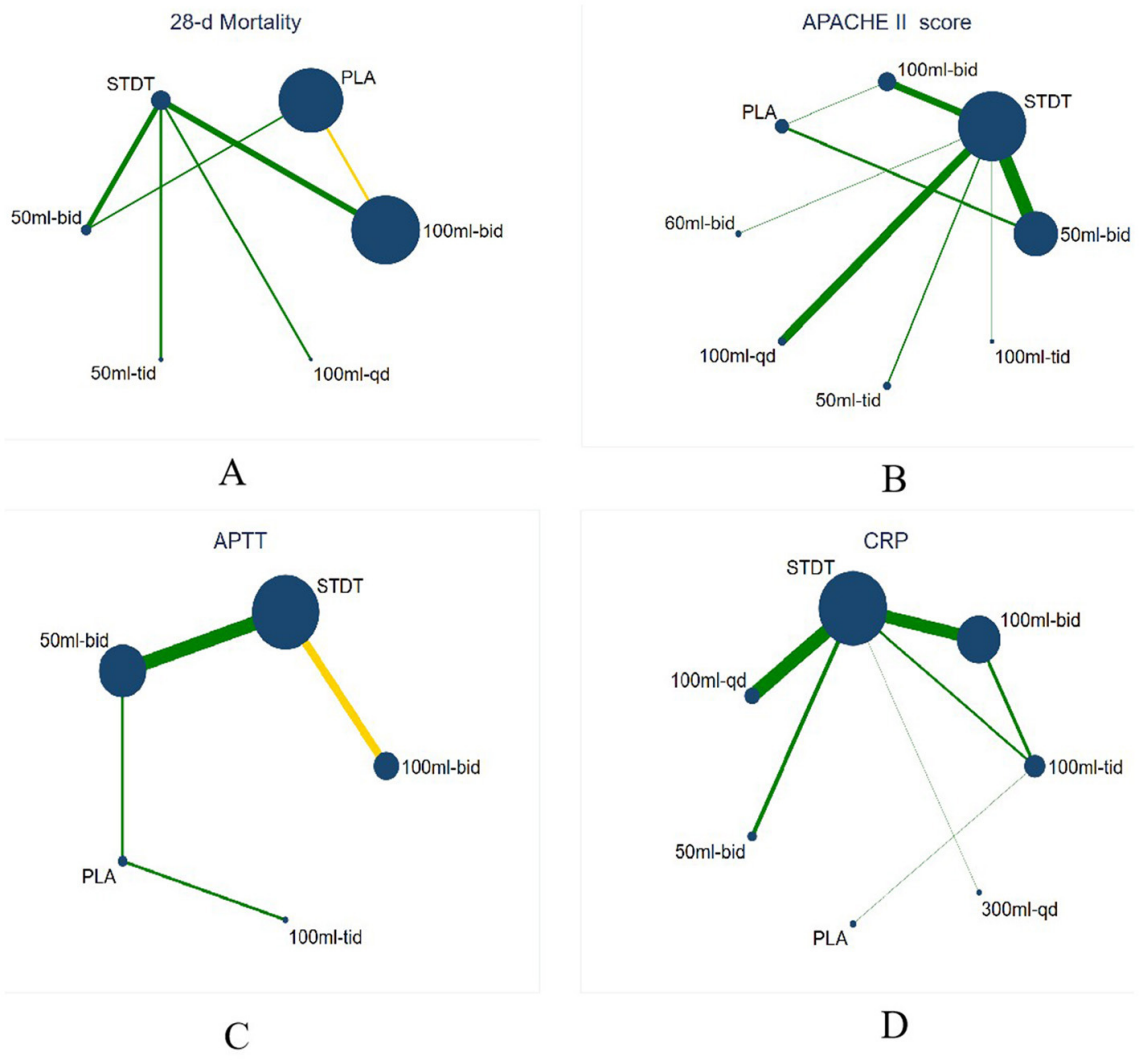
Network evidence plot. The size of the dots indicates the sample size, the larger the point, the larger the sample size, the thickness of the edges indicates the number of studies, and the yellow edges indicate that the study was blinded. **(A)** Network evidence plot for 28-d mortality, **(B)** Network evidence plot for APACHE II score, **(C)** Network evidence plot for APTT, **(D)** Network evidence plot for CRP.

**Figure 5:**
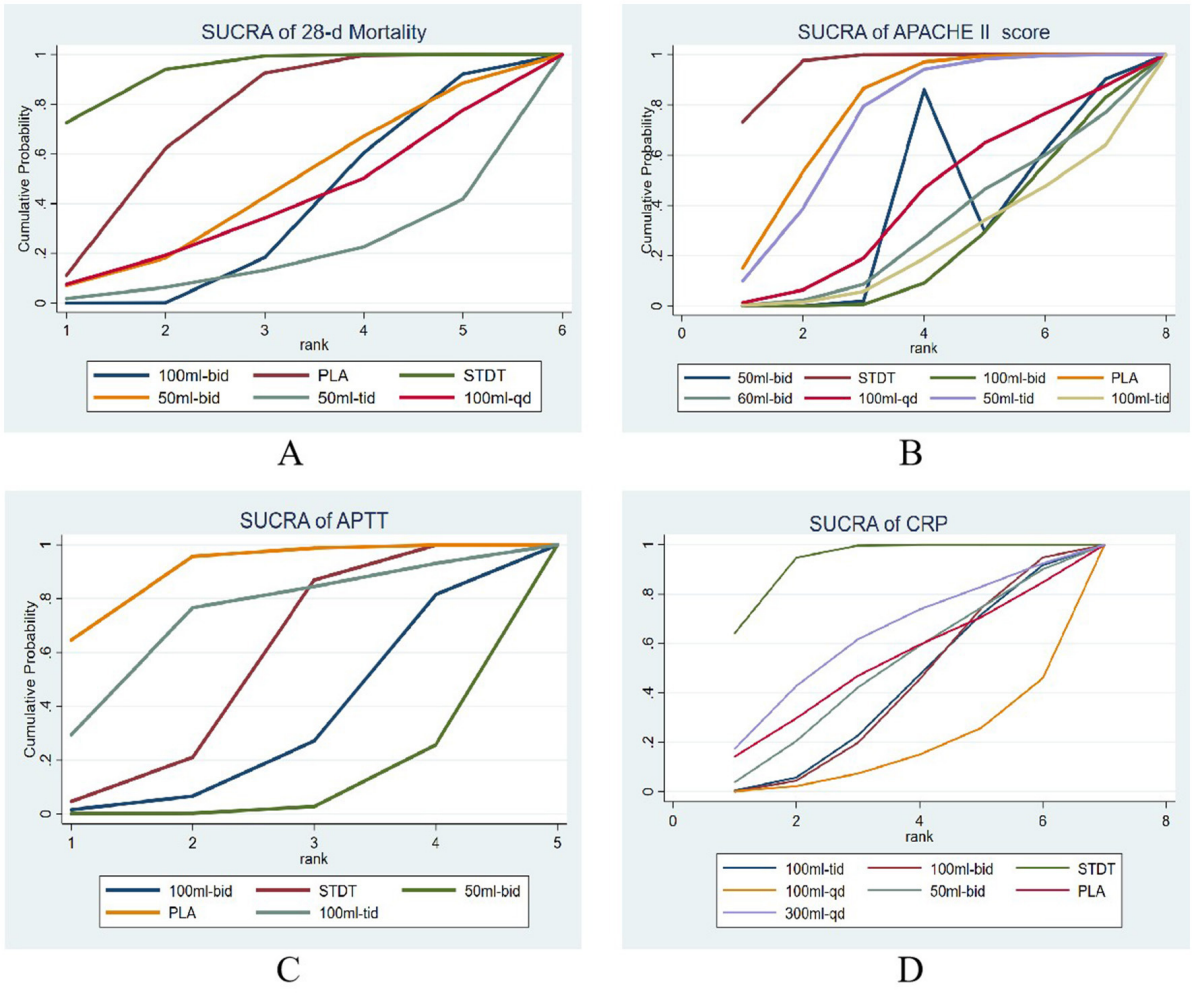
The SUCRA of the estimated probability among the treatments. **(A)** Network evidence plot for 28-d mortality, **(B)** Network evidence plot for APACHE II score, **(C)** Network evidence plot for APTT, **(D)** Network evidence plot for CRP.

The (9 RCTs, 2,646 patients) network evidence plot for 28-day mortality is shown in Figure 4A. All studies were two-arm, comprising nine studies (16, 22, 36, 40, 41, 54, 56–58). A smaller area under the curve (AUC) indicated greater efficacy in reducing 28-day mortality, as illustrated in the SUCRA plot (Figure 5A). The SUCRA table (Table 3) presents the percentage of AUC for the seven interventions. Analysis of the plot and table indicates that the efficacy of XBJ in reducing 28-day mortality is ranked as follows: STDT > PLA > 50 Milliliter (ml)-bis in die (bid) > 100 ml- bid > 100 ml-quaque die (qd) > 200 ml-bid > 50 ml-ter in die (tid). The 50 ml-tid dosage demonstrated greater efficacy in reducing 28-day mortality than other dosages, while STDT proved to be the least effective among the interventions assessed.

**Table 3:** The SUCRA.

	**SUCRA (%)**			
**Treatments**	**Outcomes**			
	**28-d mortality**	**APACHE II score**	**APTT**	**CRP**
50 ml-bid	44.7	26.2	7.1	48.3
60 ml-bid	NA	31.7	NA	NA
50 ml-tid	17.1	74.3	NA	NA
100 ml-qd	37.8	43.2	NA	16.1
100 ml-bid	34.2	25.5	29.1	39.8
100 ml-tid	NA	24.6	70.9	39.9
300 ml-qd	NA	NA	NA	61.9
STDT	93.2	95.8	53.1	93.1
PLA	73	78.7	89.7	50.9

#### 3.5.2 APACHE II score

The network evidence plot graded by APACHE II (25 RCTs, 2,200 patients) is illustrated in Figure 4B, featuring eight interventions. Three research with four arms were included (22, 47, 56), while the remaining 22 studies were all two-arm studies (19, 20, 22, 25–29, 33, 35, 37–42, 44, 50, 53–55, 59). A smaller AUC indicated a more favorable outcome for enhancing the APACHE II score, as illustrated in the SUCRA plot (Figure 5B). The SUCRA table (Table 3) presents the percentage of AUC for the eight interventions. By integrating the plot and the table, it can be inferred that the efficacy of XBJ in enhancing the APACHE II score is rated as follows: STDT > PLA > 50 ml-tid > 100 ml-qd > 60 ml-bid > 50 ml-bid > 100 ml-bid > 100 ml-tid. The 100 ml-tid was more effective in enhancing the APACHE II score than the other intervention dosages, whereas STDT was the least beneficial.

#### 3.5.3 APTT

The network evidence plot for activated partial thromboplastin time (APTT; 13 RCTs and 1,458 patients) is illustrated in Figure 4C, which includes five interventions. The literature included 13 studies (21, 29, 30, 32, 34, 35, 37, 38, 46, 48, 49, 51, 59), all of which were two-arm studies. A reduced AUC correlated with a more favorable outcome for the enhancement of APTT, as illustrated in the SUCRA plot (Figure 5C). The SUCRA table of AUC (Table 3) presents the percentage of the AUC for the five interventions. By integrating the plot and table, it can be inferred that the effectiveness of XBJ in enhancing APTT had been graded as follows: PLA > 100 mL tid > STDT > 100 ml-bid > 50 ml-bid. The 50 ml-bid dosages showed superior efficacy in enhancing APTT compared to other intervention dosages, whereas PLA exhibited the least efficacy.

#### 3.5.4 C-reactive proteins

The network evidence plot for C-reactive protein (CRP) based on 12 RCTs (889 patients) is presented in Figure 4D, with seven therapies included. The literature comprised 12 papers featuring nine two-arm studies (23, 25–27, 35, 38, 41, 43, 52) based on treatment duration, along with one six-arm research (60) and two four-arm studies (20, 24). A reduced AUC indicated superior efficacy in enhancing the CRP levels, as illustrated in the SUCRA plot (Figure 5D). The SUCRA table (Table 3) illustrates the percentage of the AUC for the seven interventions. By combining the plot and table, it can be inferred that the effectiveness of XBJ in enhancing CRP levels was rated in the following order: STDT > 300 ml-qd > PLA > 50 ml-bid > 100 ml-tid > 100 ml-bid > 100 ml-qd. The efficacy of 100 ml-qd in enhancing CRP was higher than that of the other intervention doses, whereas STDT exhibited the least efficacy.

**Figure 6:**
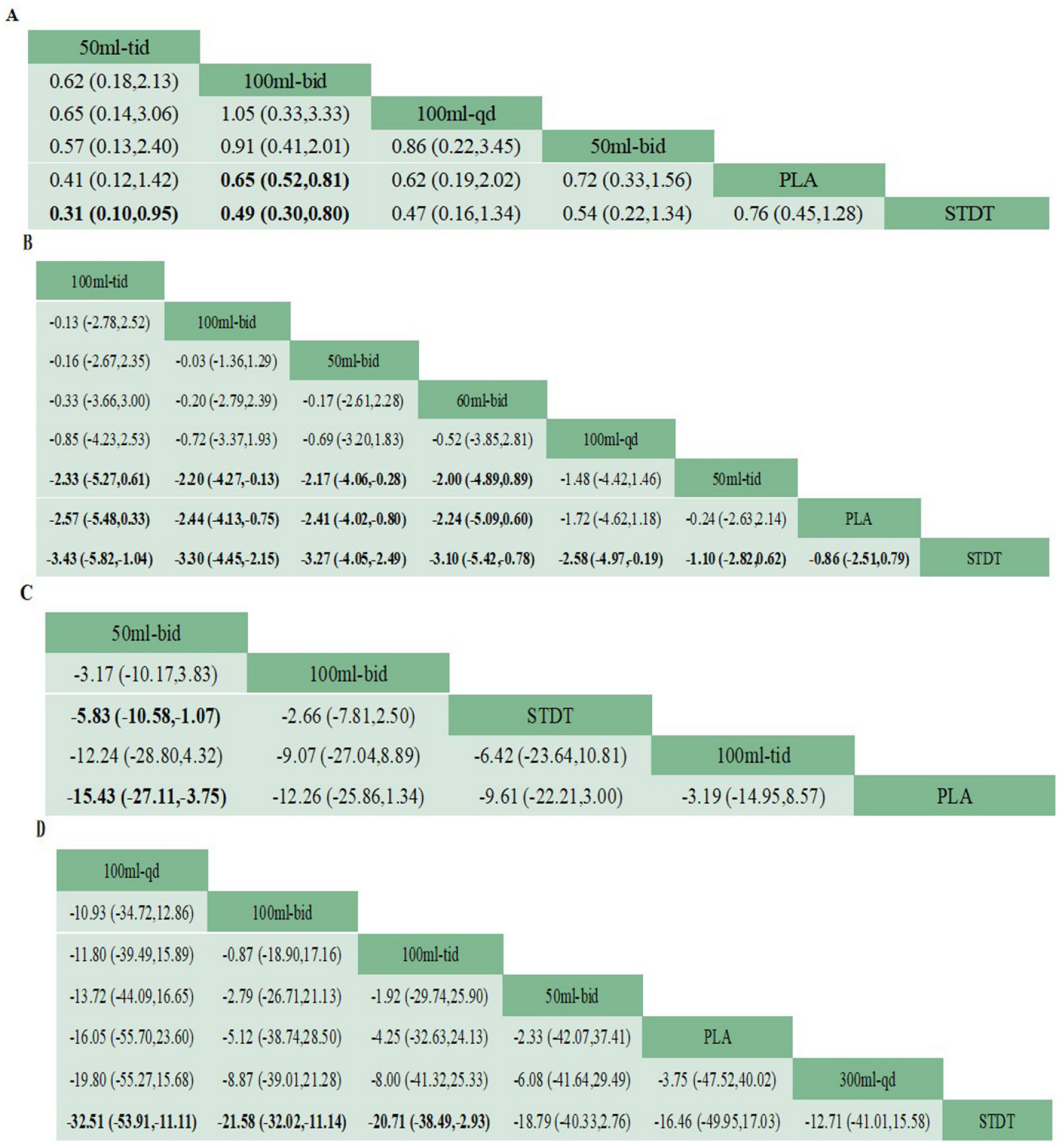
Netleague table of the four outcome indicators sorted by SUCRA values. Any statistically significant findings are highlighted in bold font. **(A)** Network evidence plot for 28-d mortality, **(B)** Network evidence plot for APACHE II score, **(C)** Network evidence plot for APTT, **(D)** Network evidence plot for CRP.

**Figure 7:**
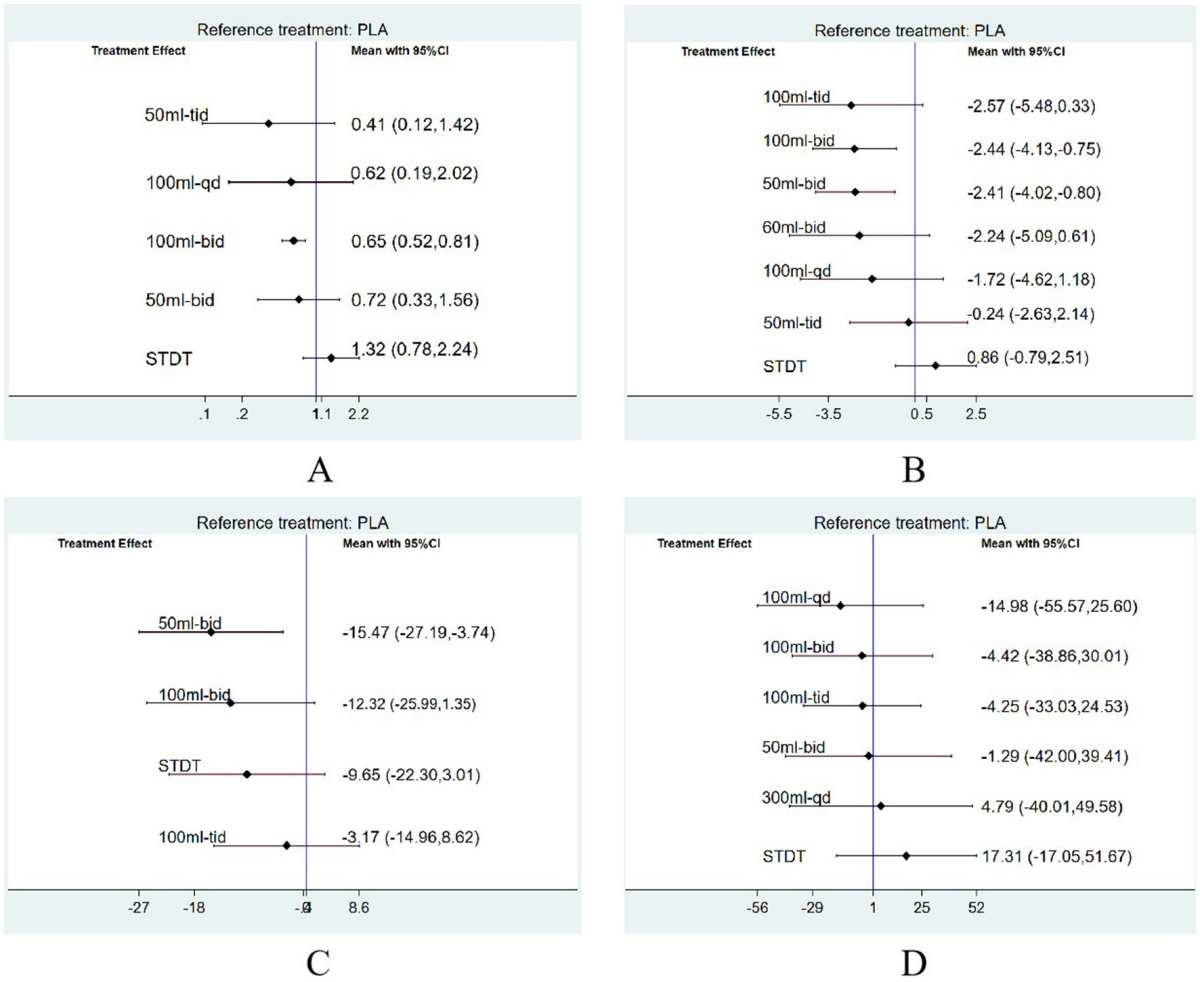
The forest plots depicting the four outcomes in comparison to the placebo were generated via frequency-based methods. **(A)** Network evidence plot for 28-d mortality, **(B)** Network evidence plot for APACHE II score, **(C)** Network evidence plot for APTT, **(D)** Network evidence plot for CRP.

A net league table of the four outcomes illustrates the results of the two-by-two comparisons between the 10 interventions shown in Figures 6A–D, with a 95% Cl excluding one indicating statistical significance. ORs of <1 indicated a preference for the intervention measures defined in the columns, and a smaller OR in the two-by-two comparisons suggested better efficacy. The forest plot in Figures 7A–D shows a comparison of the four outcomes with the placebo. A smaller OR indicated a more favorable efficacy of the intervention compared to a placebo. The OR for each intervention aligned with the findings reported in the net league table (Figure 6).

### 3.6 Publication bias

**Figure 8:**
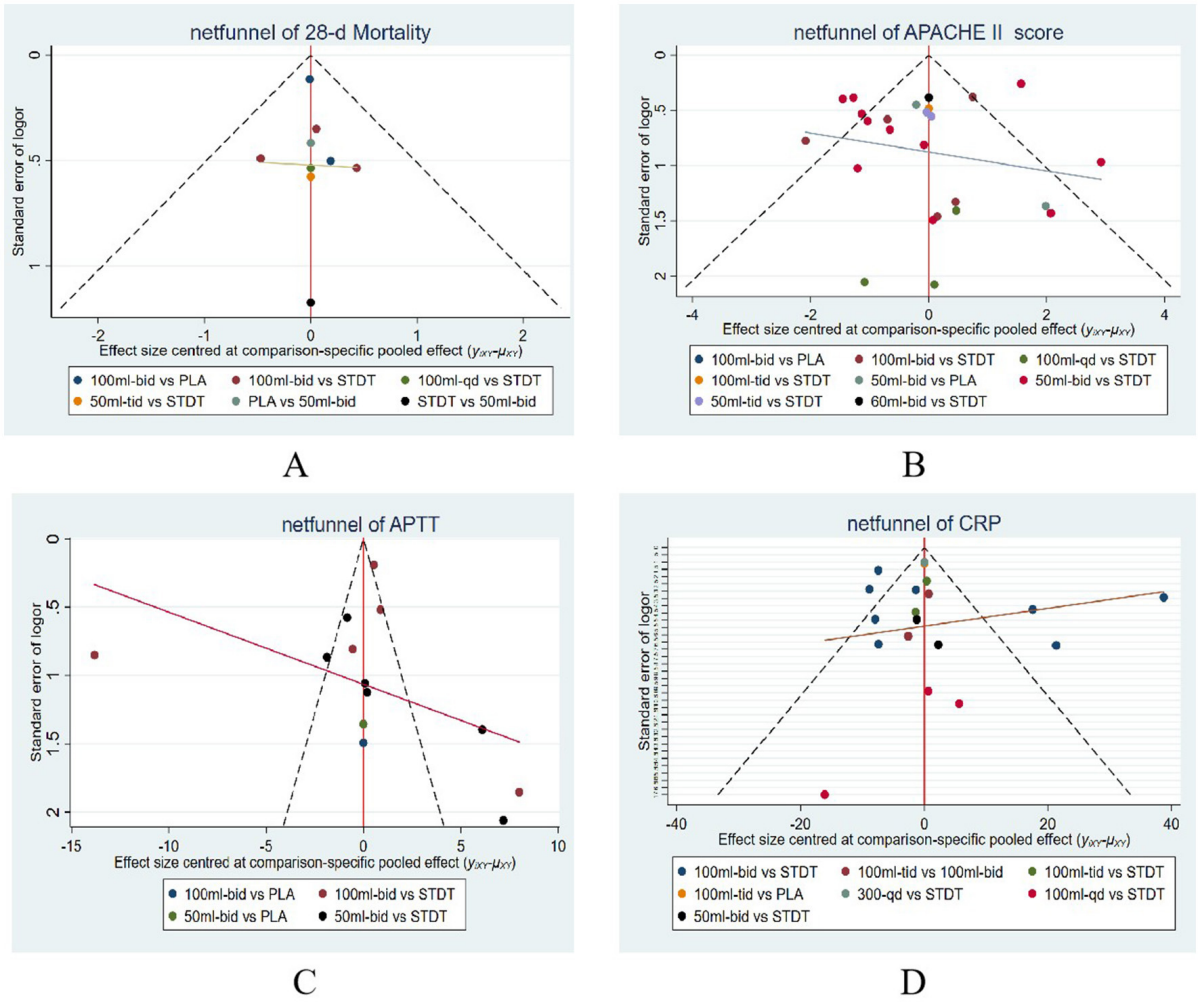
A funnel plot for evaluating bias risk. **(A)** Network evidence plot for 28-d mortality, **(B)** Network evidence plot for APACHE II score, **(C)** Network evidence plot for APTT, **(D)** Network evidence plot for CRP.

Publication bias was assessed using funnel plots generated from the four outcomes measures using Stata15 software. As shown in Figures 8A–D, the comparatively corrected funnel plots for the four outcomes demonstrated a relatively symmetrical distribution. This suggests that there was no substantial evidence of publication bias across all studies incorporated in the NMA. The detection of publication bias in the funnel plot involves a degree of subjectivity; therefore, we employed the Egger method, which identifies publication bias when the P-value is less than 0.05. Each of the four outcome indicators underwent the Egger test, resulting in a P-value of 0.218 for 28-day mortality, 0.929 for APACHE II scores, and 0.831 for APTT, indicating the absence of publication bias in all three outcome indicators. The correlation coefficient was 0.929, and the P value for APTT was 0.831, signifying the absence of publication bias for all three outcome measures. The P-value for CRP was 0.002, suggesting the presence of publication bias. We evaluated the stability of the aggregated results using the trim and fill analysis for CRP as an outcome measure. The P-value from the random-effects model employing this method was also 0.002, with a 95% confidence interval of (0, 5.496), which included 1, indicating that the results remained statistically insignificant and that there was no reversal of findings. This suggests that publication bias had minimal impact on the results, and the combined findings were relatively robust.

## 4 Discussion

Traditional Chinese medicine has evolved over the past millennia, with extensive clinical practice demonstrating its significant efficacy. The integration of Chinese medicine with Western medical practices has enhanced treatment outcomes, mitigating the adverse reactions associated with Western medicine, and bolsters patient immunity. Many proprietary Chinese medicines have been used in the treatment of sepsis, such as ShenFu injection (SF) for infectious shock, ShengMeiFang (SMF) for sepsis with deficiency of qi and yin, Xuan Bai Cheng Qi Tang (XBCQ) for sepsis with acute respiratory distress syndrome (ARDS), and Qing Wen Bai DuTang (QWBD) for sepsis with toxin-heat internalized sepsis. Furthermore, XBJ is a traditional Chinese medicine formulation used extensively in clinical adjunctive therapy for various disorders. In addition to its application in the adjuvant treatment of sepsis, numerous evidence-based studies have indicated that this injection enhances clinical efficacy and decreases mortality rates in the management of severe pneumonia (61), acute pancreatitis (62), novel coronavirus infections (63), and other conditions. The high morbidity, mortality, and rehospitalization rates associated with sepsis have become a significant medical concern in global healthcare. During the pharmacological management of sepsis, an increase in antibiotic use is correlated with an increase in the prevalence of drug-resistant and pan-resistant bacteria. This phenomenon diminishes the body's immune response, compromises its defense against pathogen invasion, significantly elevates the risk of re-infection, and contributes to an increased mortality rate associated with the disease. This study aimed to identify the optimal dose and frequency of XBJ administration to enhance the clinical efficacy of supplemental sepsis treatment.

This review included 43 RCTs, including 5,818 participants, evaluating 10 treatments with varying dosages and frequencies. The MA results indicated that XBJ at any specified doses and frequencies significantly improved the 28-day mortality, APACHE II score, APTT, and CRP levels compared to placebo and STDT. Numerous published MAs have shown that XBJ improves 28-day mortality, APACHE II scores (13, 15, 64), and CRP levels (15), corroborating the findings of this study. Furthermore, various animal experiments and network pharmacology studies have revealed that XBJ enhances coagulation function in patients with sepsis (65). Additionally, an MA revealed that XBJ not only elevated platelet levels in patients with sepsis but also reduced APTT and PT (66). thereby offering empirical support for the findings of APTT reduction in this study. A certain NMA report revealed that 50 ml-tid of XBJ was superior to other dosages in enhancing 28-day mortality, 100 ml-tid was more effective than alternative doses in improving APACHE II scores, 50 ml-bid was more efficacious than other doses in enhancing APTT, and 100 ml-qd significantly improved CRP levels. The administration of 100 ml of XBJ was more effective in enhancing APTT and CRP levels, whereas a larger daily dosage was more effective in decreasing clinical mortality (150 ml) and improving the prognosis of patients with sepsis (300 ml).

This study reviewed several prominent conventional databases and ultimately identified 43 RCTs, of which 41 were published in Chinese. Some Chinese studies lacked detailed descriptions of randomization and blinding, whereas others acknowledged these methods but failed to describe in detail their implementations. This inconsistency results in an elevated risk of bias, potentially affecting the outcomes and introducing a degree of precision bias in interpreting the results. Furthermore, the baseline characteristics in the literature were inadequately reported, for instance, while symptomatic treatment or conventional medication was referenced, the specific medications used were not disclosed. Additionally, some studies omitted data without providing explanations for the participant's dropouts. The literature indicated that a brief treatment regimen may also influence outcomes. Egger's test indicated the absence of publication bias in the outcomes of 28-day mortality, APACHE II score, and APTT. Furthermore, the findings related to the trim and fill analysis demonstrated that CRP publishing bias did not significantly influence the results, and the combined outcomes were robust.

Given that the literature lacks detailed information on the medications employed in conventional treatment, and considering that the enhancement of outcome indicators may be influenced by conventional medication, potentially skewing the interpretation of results, the SUCRA values derived from the final NMA should be interpreted with caution and should not be generalized. A single-day administration of 100 ml is most effective in enhancing APTT and CRP levels, while a 150 ml dose is superior for reducing 28-day mortality. A 300 ml dose effectively improves the APACHE II Score. Therefore, in clinical practice, the total single-day dosage of XBJ should be maintained between 100 ml and 300 ml. Furthermore, most studies are conducted over a duration of 7 days, after which varying doses and frequencies (totaling between 100 and 300 ml) are administered based on the patient's condition, yielding improved efficacy and reduced costs in clinical settings.

## 5 Conclusion

In conclusion, XBJ enhanced the 28-day mortality, APACHE II score, APTT, and CRP levels in patients with sepsis. The total dose of XBJ that enhanced APTT and CRP levels was 100 ml, and the application of 50 ml-tid and 100 ml-tid further improved the 28-day mortality and APACHE II scores. Consequently, these findings offer a reference for the clinical application of XBJ injections. This outcome requires validation through randomized controlled trials with substantial sample sizes and comprehensive experimental designs. This study provides a reference value for the therapeutic application of XBJ and offers evidence-based medical support for its clinical investigation.

## Data Availability

The original contributions presented in the study are included in the article/Supplementary material, further inquiries can be directed to the corresponding author.
