# Supplementary material for: Effects of different dosages/frequency of Xuebijing injection (a Chinese patent) for sepsis: a network meta-analysis of randomized controlled trials
Source: Front Med (Lausanne). 2025 May 22;12:1577414. doi: 10.3389/fmed.2025.1577414 (PMC12137327; doi:10.3389/fmed.2025.1577414)
Supplement: Supplementary file 1 [file Table_1.docx]

Table 4. Supplementary Appendix S1.

| Study | A sample size of patients,N | treatments | man/women | age | medication | duration | randomized and blind methods |
| --- | --- | --- | --- | --- | --- | --- | --- |
| Chen.et al^.^(20) | 78 | 50ml-bid | 24/15 | 56.51±18.10 | Bundle Therapy of Sepsis | 7d | randomized |
|  |  | PLA | 25/14 | 62.08±15.31 |  |  |  |
| Cheng.et al.(21) | 90 | 100ml-tid(q8h)(3d) | 18/12 | 50.8±11.6 | Anti-infective and associated symptomatic supportive treatment. | 3d/7d | randomized |
|  |  | 100ml-tid(q8h)(7d) |  |  |  |  |  |
|  |  | 100ml-bid(q12h)(3d) | 17/13 | 51.4±12.6 |  |  |  |
|  |  | 100ml-bid(q12h)(7d) |  |  |  |  |  |
|  |  | STDT(3d) | 19/11 | 50.0±11.5 |  |  |  |
|  |  | STDT(7d) |  |  |  |  |  |
| Dai.et al.(22) | 92 | 100ml-bid | 25/21 | 43.72±3.03 | Antimicrobial therapy,blood purification,fluid replenishment,nutritional assistance,essential organ maintenance,and respiratory support. | 7d | randomized |
|  |  | STDT | 26/20 | 42.86±32.1 |  |  |  |
| Dong.et al.(23) | 86 | 100ml-bid | 24/19 | 55.17±12.89 | Fluid  resuscitation,anti infection,vasopressor,positive inotropic drugs,sedation and analgesia,muscle relaxation,mechanical ventilation,CRRT treatment:blood glucose control;Prevention of stress ulcers;nutritional support,etc | 7d | randomized |
|  |  | STDT | 23/20 | 53.98±13.55 |  |  |  |
|  |  |  |  |  |  |  |  |
| Dou et al.(24) | 91 | 100ml-bid | 27/18 | 61.0±14.8 | Administer treatment according to with the 2014 Chinese Guidelines for the Management of Severe Sepsis and Septic Shock. | 5d | randomized |
|  |  | STDT | 29/17 | 58.3±15.6 |  |  |  |
| Gong et al.(25) | 62 | 50ml-bid | 18/13 | 62.86±12.25 | Anti infection,  fluidresuscitation,  organ maintenance | 7d | Randomized |
|  |  | STDT |  | 64.12±10.92 |  |  |  |
| Ji.et al.(26) | 79 | 100ml-tid | 25/16 | 46.73±4.92 | Standard symptomatic management of severe acute pancreatitis complicated by sepsis | 7d | Randomized |
|  |  | STDT | 21/17 | 45.91±5.02 |  |  |  |
|  |  |  |  |  |  |  |  |
| Ji.et al.(27) | 80 | 50ml-bid | 25/15 | 46.5±13.5 | Fluid  resuscitation,anti infection,Fluid replenishment,blood pressure rise,hormone therapy,and complication avoidance | 7d | randomized |
|  |  | STDT | 23/17 | 45.8±11.3 |  |  |  |
| Jia.et al.(28) | 70 | 100ml-bid(3d) | 19/16 | 47.8±9.5 | Anti infection,Antishock therapy,hormone therapy,and improved blood glucose monitoring | 3d/7d | randomized |
|  |  | 100ml-bid(7d) |  |  |  |  |  |
|  |  | STDT(3d) | 20/15 | 49.7±10.1 |  |  |  |
|  |  | STDT(3d) |  |  |  |  |  |
| Jiang.et al.(29) | 190 | 100ml-bid | 59/36 | 49.4±9.79 | Fluid  resuscitation,,anti infection,analgesia and Sedation  ,nutritional support,Respiratory assistance,preservation of internal homeostasis,and rectification of hydration and electrolyte imbalances,etc. | 7d | randomized |
|  |  | STDT | 57/38 | 49.35±9.82 |  |  |  |
| Jiang.et al.(30) | 122 | 60ml-bid(q12h) | 35/20 | 42±17 | Fluid  resuscitation,eliminate the infection cause and administer effective antibiotics;actively address the primary condition;regulate blood glucose levels;sustain electrolyte and acid-base equilibrium;provide nutritional support;and implement mechanical breathing if required. | 5d | randomized |
|  |  |  |  |  |  |  |  |
|  |  |  |  |  |  |  |  |
|  |  |  |  |  |  |  |  |
|  |  |  |  |  |  |  |  |
|  |  | STDT | 42/25 | 47±9 |  |  |  |
|  |  |  |  |  |  |  |  |
|  |  |  |  |  |  |  |  |
|  |  |  |  |  |  |  |  |
|  |  |  |  |  |  |  |  |
| Jiang.et al.(31) | 80 | 50ml-bid | 24/16 | 50.25±12.31 | Nutritional support,fluid  resuscitation,symptomatic management,  primary disease treatment | 7d | randomized |
|  |  | PLA | 25/15 | 50.1±12.22 |  |  |  |
| Li et al.(32) | 200 | 100ml-bid | 55/45 | 64.50±1.5 | Anti infection,fluid  resuscitation,nutritional support | 14d | Randomized and single-blind |
|  |  | STDT | 56/44 | 65.50±2.5 |  |  |  |
| Liu.et al.(33) | 64 | 100ml-qd | 18/16 | 47.6±16.1 | Primary disease treatment,anti infection,fluid  resuscitation,sedation and analgesia,regulation of blood glucose levels,  ,nutritional support,primary organ function support therapy | 7d | randomized |
|  |  | STDT | 16/14 | 49.6±15.9 |  |  |  |
| Liu.et al.(17) | 1817 | 100ml-bid(q12h) | 580/331 | 56.3±13.4 | Anti biotics,maintenance of arterial blood pressure with a combination of volume resuscitation and vasopressors,and early treatment of the source of infection were recom mended | 5d | randomized and double-blind |
|  |  | PLA(q12h) | 619/287 | 56.8±13.6 |  |  |  |
| Liu.et al.(35) | 142 | 50ml-bid(q12h) | 44/28 | 44．35±12．7 | Primary disease treatment,anti biotics,organ function protection and nutritional support | 7d | randomized |
|  |  | STDT | 39/31 | 42．8士13．5 |  |  |  |
| Lu et al.(35) | 78 | 50ml-bid | 21/17 | 59.8 | Fluid resuscitation,anti biotics,nutritional support,Insulin regulates blood glucose levels. | 7d | randomized |
|  |  | STDT | 19/20 | 61.4 |  |  |  |
| Ma et al.(36) | 136 | 50ml-bid | 34/34 | 64.68±15.68 | Primary disease treatment,anti biotics,organ function protection | 7d | randomized |
|  |  | STDT | 38/30 | 66.13±15.83 |  |  |  |
| Ming.et al.(37) | 60 | 300ml-qd | 38/22 | 43±25 | Fluid resuscitation,anti biotics,stabilize the internal environment and facilitate symptomatic management. | 7d | randomized |
|  |  | STDT |  |  |  |  |  |
| Pu.et al.(38) | 90 | 50ml-tid(7d) | 24/21 | 85.88±6.85 | Primary disease treatment,anti inflammatory drug therapy,fluid resuscitation,nutritional support,rectify diseases of water,electrolyte,and acid-base equilibrium. | 7d/14d | randomized |
|  |  | 50ml-tid(14d) |  |  |  |  |  |
|  |  | STDT(7d) | 23/22 | 83.17±5.92 |  |  |  |
|  |  | STDT(14d) |  |  |  |  |  |
| Shao.et al.(39) | 64 | 50ml-bid | 21/18 | 55.7±15.3 | Bundle therapy is provided in accordance with the 2004 International Campaign to Save Sepsis(SSC)guidelines. | 7d | Prospective randomized controlled |
|  |  | STDT | 12/13 | 56.2±15.5 |  |  |  |
| Shao.et al.(40) | 63 | 100ml-bid(q12h) | 44/19 | 72.71 | fluid resuscitation,vasoactive drugsand anti biotics,early recovery of gastrointestinal function,intensified insulin therapy,etc | 7d | randomized |
|  |  | STDT |  |  |  |  |  |
| Shen et al.(41) | 76 | 100ml-bid | 22/16 | 52.65±7.48 | Anti biotics,regulate blood pressure and glucose levels  ,rectify diseases of water,electrolyte,and acid-base equilibrium,nutritional support,mechanical ventilation，primary disease treatment. | 14d | randomized |
|  |  | STDT | 20/18 | 51.87±7.75 |  |  |  |
| Shi et al.(42) | 70 | 100ml-qd | 18/17 | 53.78±8.94 | Mechanical ventilation,anti biotics,regulate glucose levels,nutritional support,rectify diseases of water,electrolyte,and acid-base equilibrium. | 7d | randomized |
|  |  | STDT | 19/16 | 54.57±8.51 |  |  |  |
| Song et al.(43) | 94 | 50ml-bid | 35/12 | 57.8±6.2 | Anti biotics,fluid resuscitation,hemodynamic support. | 7d | randomized |
|  |  | STDT | 30/17 | 61.3±5.4 |  |  |  |
| Su.et al.(44) | 62 | 100ml-bid | 19/11 | 68.4±7.8 | Anti biotics,mechanical ventilation,fluid resuscitation,regulate glucose levels,nutritional support,primary disease treatment,support for organs  ,symptomatic treatment. | 7d | randomized |
|  |  | STDT | 17/15 | 65.1±10.6 |  |  |  |
| Sun.et al.(45) | 80 | 100ml-bid(q12h) | 58/22 | 46±25 | primary disease treatment,anti biotics,nutritional support and preserve internal ecological equilibrium. | 7d | randomized |
|  |  | STDT |  |  |  |  |  |
| Sun.et al.(46) | 84 | 50ml-bid | 25/17 | 43.66±6.98 | Nutritional support,support for organs,anti biotics,standard wound debridement and dressing alteration. | 7d | randomized |
|  |  | STDT | 23/19 | 45.11±7.24 |  |  |  |
| Wang.et al.(47) | 80 | 100ml-qd | 29/11 | l6～52 | Anti biotics,fluid resuscitation,Nutritional support,  support for organs. | 14d | randomized |
|  |  | STDT | 28/12 | l8～55 |  |  |  |
| Wang.et al.(48) | 62 | 100ml-qd | 16/15 | 51.21±18.07 | Anti biotics,fluid resuscitation,use of vasoactive drugs and mechanical ventilation. | 7d | randomized |
|  |  | STDT | 15/16 | 51.33±16.98 |  |  |  |
| Wang et al.(49) | 90 | 100ml-bid | 28/17 | 53.01±9．24 | Preserve electrolyte equilibrium and administer intravenous nourishment,  anti biotics,fluid resuscitation,regulate glycemic levels,use of vasoactive drugs. | 7d | randomized |
|  |  | PLA | 25/20 | 52．59±8．97 |  |  |  |
| Wu.et al.(50) | 62 | 50ml-bid | 17/15 | 50.96±15.08 | Primary disease treatment,anti biotics,fluid resuscitation,nutritional support and support for organs. | 7d | randomized |
|  |  | STDT | 16/14 | 52.35±15.9 |  |  |  |
| Xie.et al.(51) | 98 | 50ml-bid | 25/24 | 53.4±12.3 | Anti biotics,fluid resuscitation,nutritional support,surgical intervention,breathing assistance,  use of vasoactive drugs. | 14d | randomized |
|  |  | STDT | 23/26 | （52.8±11.5 |  |  |  |
| Xing et al.(52) | 63 | 100ml-bid(7d) | 24/9 | 47.82+21.98 | Primary disease treatment,anti biotics,breathing assistance,preserve hemodynamic stability,nutritional support. | 7d/10d | randomized |
|  |  | 100ml-bid(10d) |  |  |  |  |  |
|  |  | STDT(7d) | 20/10 | 58.63+21.25 |  |  |  |
|  |  | STDT(10d) |  |  |  |  |  |
| Xu.et al.(53) | 64 | 50ml-bid | 18/16 | 50.42±8.92 | Implement interventions in accordance with the sepsis treatment protocols established by the American Society for Critical Care Medicine. | 7d | randomized |
|  |  | STDT | 16/14 | 52.06±9.05 |  |  |  |
| Yang et al.(54) | 102 | 50ml-bid | 60/42 | 52.19±  10.67 | Anti biotics and support for organs | 14d | randomized |
|  |  | STDT |  |  |  |  |  |
| Yin et al.(55) | 171 | 100ml-bid | 59/29 | 59(53–74) | Anti biotics,fluid resuscitation,mechanical ventilation,vasoac tive agents,nutritional support,and transfusion of blood products,whereas coagulation inhibitors and anticoagulants werenotusedinourEICU | 7d | Retrospective study |
|  |  | STDT | 51/32 | 56(49–73) |  |  |  |
| Zhang.et al.(56) | 120 | 100ml-bid | 64/56 | 58.41±l7.6 | Anti biotics,hormones,regulation of water-electrolyte and acid-base equilibrium and more standard therapies. | 7d | randomized |
|  |  | STDT |  |  |  |  |  |
| Zhang.et al.(57) | 300 | 50ml-bid | 79/71 | 5.16±9.29 | Administer consistent therapy in accordance with the sepsis treatment guidelines. | 7d | randomized |
|  |  | STDT | 67/83 | 75.56±8.36 |  |  |  |
| Zhang et al.(58) | 90 | 100ml-bid | NA | 56.9±13.5 | Anti biotics,regulate blood glucose,  nutritional support,regulation of water-electrolyte and acid-base equilibrium and mechanical ventilation. | 7d | randomized |
|  |  | STDT |  | 58.2±12.7 |  |  |  |
| Zhang et al.(59) | 86 | 50ml-bid | 23/20 | 40.16±3.58 | Fluid resuscitation,use of vasoactive drugs,anti biotics,protecting renal function,offering dietary assistance,regulating hydration,electrolyte balance,acid-base equilibrium,and addressing underlying conditions. | 14d | randomized |
|  |  | STDT | 22/21 | 41.28±3.06 |  |  |  |
| Zhang et al.(60) | 64 | 50ml-bid | 19/13 | 50.33+12.47 | Primary disease treatment,anti-inflammatory and anti-infective  blood transfusion and fluid replacement,mechanical ventilation and analgesia and sedation,,regulate blood glucose  ,nutritional support,preserve water and electrolyte equilibrium,etc. | 7d | randomized |
|  |  | STDT | 23/9 | 49.64+11.92 |  |  |  |
| Zhu et al.(61) | 66 | 100ml-bid | 39/27 | 42±11 | Primary disease treatment,anti biotics,support for organs.,preserve water and electrolyte equilibrium,nutritional support. | 7d | randomized |
|  |  | PLA |  |  |  |  |  |
